# Optimization of Imminent Labor Prediction Systems in Women with Threatened Preterm Labor Based on Electrohysterography

**DOI:** 10.3390/s21072496

**Published:** 2021-04-03

**Authors:** Gema Prats-Boluda, Julio Pastor-Tronch, Javier Garcia-Casado, Rogelio Monfort-Ortíz, Alfredo Perales Marín, Vicente Diago, Alba Roca Prats, Yiyao Ye-Lin

**Affiliations:** 1 Centro de Investigación e Innovación en Bioingeniería, Universitat Politècnica de València, 46022 Valencia, Spain; jupastro@etsii.upv.es (J.P.-T.); jgarciac@ci2b.upv.es (J.G.-C.); yiye@ci2b.upv.es (Y.Y.-L.); 2 Servicio de Obstetricia, Hospital Universitario y Politécnico de La Fe, 46026 Valencia, Spain; monfort_isaort@gva.es (R.M.-O.); Perales_alf@gva.es (A.P.M.); diago_vicalm@gva.es (V.D.); roca_alb@gva.es (A.R.P.)

**Keywords:** electrohysterogram, uterine myoelectrical activity, tocolytic therapy, random forest, extreme learning machine, K-nearest neighbors, imminent labor prediction

## Abstract

Preterm birth is the leading cause of death in newborns and the survivors are prone to health complications. Threatened preterm labor (TPL) is the most common cause of hospitalization in the second half of pregnancy. The current methods used in clinical practice to diagnose preterm labor, the Bishop score or cervical length, have high negative predictive values but not positive ones. In this work we analyzed the performance of computationally efficient classification algorithms, based on electrohysterographic recordings (EHG), such as random forest (RF), extreme learning machine (ELM) and K-nearest neighbors (KNN) for imminent labor (<7 days) prediction in women with TPL, using the 50th or 10th–90th percentiles of temporal, spectral and nonlinear EHG parameters with and without obstetric data inputs. Two criteria were assessed for the classifier design: F1-score and sensitivity. RF_F1_2_ and ELM_F1_2_ provided the highest F1-score values in the validation dataset, (88.17 ± 8.34% and 90.2 ± 4.43%) with the 50th percentile of EHG and obstetric inputs. ELM_F1_2_ outperformed RF_F1_2_ in sensitivity, being similar to those of ELM_Sens_ (sensitivity optimization). The 10th–90th percentiles did not provide a significant improvement over the 50th percentile. KNN performance was highly sensitive to the input dataset, with a high generalization capability.

## 1. Introduction

Preterm labor is defined as natural or induced labor prior to 37 weeks of gestation [1]. Currently, preterm birth and its consequences are a serious problem in health systems in most developed countries, and despite the fact that the treatment protocols for these deliveries have evolved considerably and reduced the mortality of neonates, it is still associated with 35% of newborn deaths [2]. It has not been possible to reduce the prevalence of preterm births, which continues to increase year after year, assuming between 8% and 13% of total deliveries worldwide, affecting some 15 million families [3]. This increase is fundamentally associated with a more advanced mean age of pregnant woman and the increased use of fertility treatments, resulting in a higher risk of preterm delivery. Preterm deliveries increase health spending not only due to the immediate treatment required by preterm infants, but also due to normally chronic problems that tend to develop due to early deliveries, ranging from respiratory, gastrointestinal and immune problems to more severe neurological, cognitive and motor problems [4].

Prompt preterm labor diagnosis is vitally important to be able to administer uterine inhibitory drugs in time so that the corticosteroids can accelerate fetal lung development, reduce perinatal and neonatal death risk, intra ventricular hemorrhage, and underdevelopment in childhood [5]. Although great efforts have been made and obstetrical parameters such as cervical length (CL) by themselves or in combination with other biochemical markers such as fetal fibronectin (fFN) have shown a certain degree of usefulness in detecting preterm births, currently there is no technique that allows assessing time-to-delivery objectively and accurately and whether or not it will be premature [6]. A study measuring fFN and CL in women between 22 and 30 weeks’ gestation showed an AUC of 0.59 in preterm birth prediction, an AUC of 0.67 for cervical length alone and a very similar value for the combination of both [7]. Although cervical length and fFN have high negative predictive values, their positive predictive values are lower and do not identify the patients who are actually going to deliver prematurely [8].

The problem not only lies in premature births but also in the threat of premature labor (TPL), this being the most common cause of hospitalization in the second trimester of pregnancy. This involves prolonged clinical stays, drug treatment with possible side effects, significant distress for the pregnant woman and her family, reduced care for her other children (if any) and a high economic cost derived from the hospitalization and absence of the pregnant woman from work [9]. As the literature reports that only 34% of the women who go to the emergency room with threatened preterm labor give birth preterm [10], predicting whether a woman with TPL will give birth prematurely can help to optimize labor management, allowing decisions to be made that lead to the best result from the point of view of maternal-fetal health and hospital resource management [9].

The studies in the literature state that the mechanisms that trigger labor start several days or even weeks before it and involve changes in the electrical potential of myometrial muscle cells. This myoelectrical activity, known as an electrohysterogram (EHG), can be recorded on the surface of the abdomen. The bursts of action potentials in the EHG is associated with uterine contractions [11,12]. The EHG has been proposed as a promising technique for preterm labor prediction due to the fact that uterine activity increases its intensity and synchronization near labor [13,14,15]. The increased number of myometrial cells recruited in uterine contractions when labor is near results in a greater EHG amplitude, while the intensified cell excitability shifts the EHG spectral content to higher frequencies [16,17], and the labor onset entails changes in myometrial cell connectivity that modifies the regularity of the measured EHG signal: EHG predictability increases while signal complexity is reduced [18,19,20].

Labor or preterm labor prediction algorithms have been developed with over 90% accurate metrics [21,22,23,24] when using EHG recordings from women during clinical checkups under physiological conditions. However tocolytic drugs are usually administered to inhibit uterine contractions at the first sign of threatened preterm labor and modify the EHG features [25,26]. The feasibility of imminent preterm labor prediction in women with TPL undergoing tocolytic treatment by means of an artificial neural network (ANN) algorithm has been proved using median values of EHG parameters in 120 s analysis windows and obstetric data inputs [27]. Despite being one of the most frequently used classification algorithms, ANN has certain drawbacks, such as a low learning speed associated with the backpropagation algorithm and the fact that it can easily get into local optima [28]. In the present study we focus on three different computationally efficient algorithms, random forest (RF), extreme learning machine (ELM) and K-nearest neighbors (KNN), which have been used in biological classification problems to distinguish between gestational or labor contractions or term/preterm deliveries from EHG recordings from routine checkups [28]. A recent study revealed that the 90th and 10th percentiles of the EHG parameters computed in 120 s whole analysis windows outperformed median values in discerning between different obstetric scenarios, as term vs. preterm labor, in recordings from routine checkups [20]. The better ability of the 10th–90th percentiles of EHG parameters to discriminate were associated with being more representative of the contractile segments in the recording session [20], choosing the 10th or 90th percentile of the EHG parameters according to their expected trend as delivery approaches [20]. In addition, the optimization criterion in the classifier design was not clearly indicated in the literature regarding term/preterm birth or labor/nonlabor prediction, regardless of the classification algorithm employed [24,29,30,31]. In applications such as the prediction of preterm labor or imminent delivery in women with TPL, it is of vital importance to develop prediction systems with high sensitivity values.

The aim of the present work was therefore to assess and optimize the performance of EHG based computationally efficient classification algorithms (RF, ELM and KNN) for imminent labor (<7 days) prediction in women with TPL. It was also intended to determine whether the 10th–90th percentiles of EHG parameters computed in 120 s analysis windows with or without obstetric input features contain more information for imminent labor prediction in women with TPL than median values, and to determine how the optimization criteria in the classifier design affect their performance.

## 2. Materials and Methods

### 2.1. EHG Database and Characterization

A database of 140 30-min EHG recordings conducted on 84 singleton-pregnant women with TPL symptoms such as uterine dynamics and/or cervical effacement or dilatation taken at the “Hospital Universitari i Politècnic la Fe” (Valencia, Spain) from 2015 to 2019 was used in the study, which was approved by the hospital’s Institutional Review Board. Women with preterm membrane rupture were excluded. The women were informed of the aims of the study and asked to give their written consent. Most EHG signals were recorded during or after the administration of the Atosiban tocolytic agent, which blocks uterine contractility. Thirty records in the database were from women who gave imminent birth (time to delivery (TTD) ≤ 7 days) and 110 gave birth in more than 7 days. For the TTD ≤ 7 days group, 13 recordings were performed during tocolytic treatment, 13 post-tocolytic treatment and four were obtained before tocolytic treatment. In the TTD > 7 group, 47 records were taken during tocolytic treatment, 43 post-tocolytic treatment and 20 before tocolytic treatment. Obstetrical data picked up were gestational and maternal age, cervical length at the recording, parity, gestations, and abortions. This information can be found in [27].

A single bipolar EHG signal was recorded as described in [27] by means of two disposable Ag/AgCl electrodes (3M red dot 2560, wet with solid hydrogel) placed on the abdomen over the navel symmetrically to the median axis with an interelectrode distance of 8 cm (electrodes M1 and M2). Two additional electrodes were placed on the patient’s hips acting as ground and reference electrodes. Signals were digitized with a 24 bit ADC at 500 Hz and downsampled to 20 Hz. A bipolar recording was computed as the difference of the two monopolar recordings from M1 and M2 in the 0.1–4 Hz bandwidth to diminish common-mode interference. Signal segments with motion artefacts or with considerable respiratory interference were discarded in a visual inspection by experts in a double-blinded process. The EHG characteristics were worked out in 120 s windows with a 50% overlap, and were a tradeoff between saving representative sections of the recordings and a rational computational cost [25]. This avoids burst annotation and is more appropriate for future ‘real-time’ applications, bringing EHG closer to clinical practice.

Twenty-three temporal, spectral, complexity and regularity parameters were worked out in the whole EHG bandwidth, 0.1–4 Hz (unless otherwise noted), to characterize the recordings (see Table 1). Amplitude in temporal domain tends to increase as labor approaches due to the growth of cells recruited in uterine contractions [21,22]. Spectral parameters are used to assess the shift of EHG spectral content to higher frequencies as labor approaches [12,17,32]. We computed the dominant frequencies in the bandwidth 0.2–1 Hz (DF1) and in 0.34–1 Hz (DF2), considering that the Fast Wave Low of the EHG ranges from 0.2 to 0.34 Hz and Fast Wave High, which includes EHG components above 0.34 Hz, limiting the high frequency to 1 Hz to diminish ECG and respiration interference [13,33]; the high-to-low frequency energy ratio (H/L ratio, as the ratio of energy in 0.34–1 Hz to energy in 0.2–0.34 Hz); deciles of the power spectrum and the spectral moment ratio (SMR) [20,34].

A set of representative complexity and regularity parameters to distinguish between preterm and term labor were also calculated to characterize the EHG signal, as in the related literature [20]: Lempel-Ziv (Binary and multistate n = 6), which assesses signal complexity from the number of different patterns in the signals [35]; sample, fuzzy and spectral entropy, which measure regularity based on the self-similarity in temporal and spectral domains [36,37,38,39]; and time reversibility [40], which estimates the similarity of a time series when time goes forward or back. A Poincare plot [41] of consecutive EHG signal samples was obtained and parameters SD1, SD2 were also computed to assess corresponding short- and long-term EHG changes, and SD1/SD2 to measure EHG randomness [42]. To avoid redundant information, other nonlinear parameters were not calculated as in [31,43].

To obtain representative values for each EHG parameter in each recording we worked out the 50th (median) and 10th–90th percentiles of the parameters for all the analyzed windows [20]. The 50th percentile mainly assesses basal activity rather than uterine contractions in nonlabor recordings. This is because, considering uterine electrophysiology throughout pregnancy, during the 30-min recording session the time-percentage of EHG-bursts (associated with uterine contraction) is expected to be relatively low, especially in pregnancy recordings (maximum contraction rhythm: 3 in 10-min during active phase of labor). To characterize uterine contractions (EHG-Bursts) the 10th and 90th percentiles of all the analyzed windows were thus calculated. For EHG parameters with an increasing trend in contractile periods as labor approaches, as amplitude, DF1, DF2, deciles and H/L and time reversibility, the 90th percentile was computed. In contrast, for the EHG parameters whose values decrease in contractile periods as labor approaches the 10th percentile was worked out, as was the case of SMR, Binary and Multistate Lempel-Ziv (n = 6), Sample Entropy, Fuzzy Entropy, Spectral Entropy, SD1, SD2 and SD1/SD2.

### 2.2. Classifiers Design and Assessment

**Figure 1:**
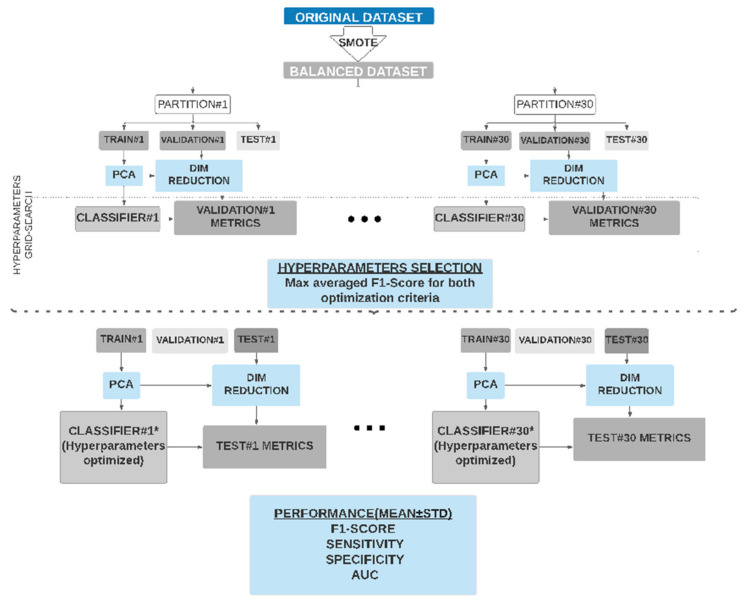
Scheme of the method used to train, validate and test the imminent labor prediction classifiers (time to delivery (TTD ≤ 7) based on EHG in women with threatened preterm labor. This was performed with two optimization criteria in the classifier design: F1-score and sensitivity.

The database was clearly imbalanced towards the patients who gave birth after 7 days (TTD ≤ 7 days 21.4%, TTD > 7 days 78.6%). To overcome this well-known problem of imbalanced data training, which may induce a clear bias towards the majority class predictions, the synthetic oversampling technique (SMOTE) [44] was used. This technique has been broadly used to deal with imbalanced classification problems [21,31,32,45]. SMOTE was applied by using five neighbors to interpolate the minority samples in a ratio of 3:1. This led to a balanced database with 110 recordings from the majority class and 90 recordings from the minority class (TTD ≤ 7 days 47%, TTD > 7 days 53%). To check the robustness of the classifiers under different data conditions a holdout method was applied 30 times by randomly splitting the database into training, validation and test subsets each time. The main purpose of repeating each experiment 30 times was to be able to reduce the possible bias induced by a particular distribution of the subsets and to ensure the strength of the statistical tests (nonparametric Wilcoxon paired test) performed to optimize the hyperparameters, based in statistically relevant differences in the validation subset. For each experiment repetition (partition), the subset percentages were distributed as follows: 1/3 for test, so as to evaluate the classifiers’ generalization capacity, and 2/3 for the classifiers’ design, training (2/3 * 2/3) and validation (2/3 * 1/3). The percentages were thus 44% training (89 samples), 23% validation (45 samples) and 33% test (66 samples), see Figure 1. For each subset (train, validation and test) we maintained the proportion of TTD ≤ 7 and TTD > 7 data, 47% of the minority class and 53% majority class. The same 30 partitions were conserved over all “stages” in the classifiers design (train and validation) and test. No randomness was involved in the case of KNN classifier training, but ELM initial weights and RF random sampling and feature selection were dealt with by initializing each classifier with the same hyperparameter combination 30 times and different fixed seeds (corresponding to 100 initializations). Trained algorithms were assessed under the validation subset and the best seed was chosen and stored as an “additional” hyperparameter, ensuring reproducibility and consistency between different trials. This seed acted in RF by forcing the algorithm to select a similar subset of features in each trial and in ELM by forcing the initial layer weights to be the same over different trials. Therefore, besides maintaining the same 30 partitions for all classifiers, for the ELM and RF ones the same combination of seeds and weights optimized in validation were used for the test. To avoid overfitting problems due to the number of input features being larger than the number of recordings (23 EHG and six obstetric), a principal component analysis (PCA) was carried out retaining 98% variance but reducing the number of input parameters [23,37,46].

The performance of three computationally efficient classification algorithms was assessed in this study: the extreme learning machine (ELM), K-nearest neighbors (KNN) and random forest (RF), all of these implemented on public packages of R. The public Ranger package for RF classifiers was used to develop random forest. This is an efficient parallel implementation of the RF algorithm proposed by Breiman in [47,48]. The number of trees, the maximum depth of these trees and the cost of division based on the criterion of gain of information were optimized. The elmNNRcpp package, based on the implementation proposed by Huang was selected for the ELM classifier [49]. The hyperparameters to be optimized were in this case the number of neurons in the hidden layer, and the activation function. KNN was implemented in the R KNN algorithm, which uses the Minkowski distance and a weighting based on a probabilistic kernel [50]. The hyperparameters to optimize were number of neighbors and the Kernel used for weighting the distances. In an appendix to this article we included four tables (Table A1, Table A2, Table A3 and Table A4) detailing the gridsearch carried out for the parameters and the optimized values for the classifiers.

As previously mentioned, as classifiers with high sensitivity values are required for preterm labor or imminent delivery prediction in women with TPL, we dealt with two different optimization criteria for the classifiers’ design, the F1-Score (harmonic mean of precision and recall) and sensitivity [51]. In both cases we carried out a hyperparameter grid search (see Figure 1) and after obtaining the metrics of the classifiers in each of the 30 partitions, they were averaged, choosing the hyperparameters which gave the highest mean F1-Score in validation subsets (for both criteria). This was decided because F1 would avoid the overdetection of false positives, as happens today in clinics, where uterine inhibitors are administered to all the women with TPL symptoms. After selecting the optimal hyperparameters, their performance in the test data was assessed. For each classifier four sets of input features were appraised: (1) the 10th–90th percentiles of EHG parameters and obstetric data; (2) the 50th percentile of EHG data and obstetric data; (3) the 10th–90th percentiles of EHG parameters; (4) the 50th percentile of EHG data. Table 2 summarizes the classifiers developed according to their input dataset and optimization criterion, F1-score or sensitivity.

To assess the models’ performance, a set of metrics (F1-score, sensitivity, and specificity) was obtained for each partition in training, validating and testing the data. They were computed as follows:

(1) $$F1-score\;\left(\%\right)=\frac{2*TP}{2*TP+FP+FN}\cdot 100\;$$

(2) $$Sensitivity\;\left(\%\right)=\frac{TP}{\left(TP+FN\right)}\cdot 100\;$$

(3) $$Specificity\;\left(\%\right)=\frac{TN}{\left(TN+FP\right)}\cdot 100\;$$

where, *TP*, *TN*, *FP*, and *FN* are true positives, true negatives, false positives, and false negatives, respectively. In this work, a true positive is labor TTD ≤ 7 days correctly predicted by the algorithm. The Wilcoxon signed rank test was used to check for any statistically significant differences between pairs of classifier metrics. This was done first for all classifier metrics from the validation dataset to find any statistically significant differences for the same classifier when changing the optimization criteria (e.g., ELM_F1_2_ vs. ELM_SENS_2_). Secondly, we looked for any statistically significant differences regarding the classifier input dataset (e.g., ELM_F1_2_ vs. ELM_F1_1_) in the optimization criteria and metrics. It should be noted that validation and test results were not mixed in any case. The coefficient of variation of the above-mentioned metrics were worked out for the 30 test datasets to analyze the strength of the algorithms when using new and different sets. Finally, the receiver operating curve (ROC) was obtained and represented for the classifier with the best performance.

## 3. Results

Regarding the nature of the classifiers used in the present study, as the metrics obtained for the training subset, for which the classifiers were trained, were in most cases 100% they were not considered relevant for our purpose and are not included here.

### 3.1. Random Forest (RF)

**Figure 2:**
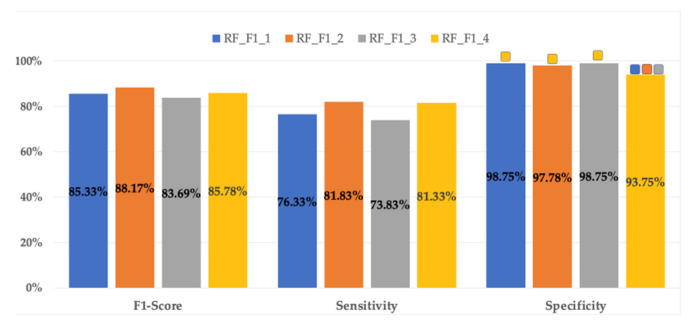
Mean values of different RF classifier metrics for validation datasets in the 30 data partitions optimized by F1-score. The same results were obtained when optimizing by sensitivity. For each metric the significant differences (*p* < 0.05) for each input dataset are marked with: 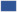 10th–90th percentiles of EHG parameters + obstetric input data; 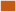 50th percentile of EHG + obstetric input data; 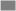 10th–90th percentiles of EHG parameters; 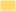 50th percentile of EHG parameters.

Regardless of the optimization criterion (F1-score or sensitivity), the best classifiers for each data input set showed the same optimal hyperparameters, that is: RF_F1_1_ = RF_SEN_1_; RF_F1_2_ = RF_SEN_2_; RF_F1_3_ = RF_SEN_3_; RF_F1_4_ = RF_SEN_4_. Figure 2 shows a bar plot of the metrics of the RF classifiers in the validation dataset for each set of input features. It can be seen in this figure that the highest mean F1-score is for RF_F1_2_ (88.17 ± 8.34%), which also has the highest sensitivity (81.83 ± 12.9%). As for RF classifiers, adding obstetric data to EHG parameters as data inputs slightly improves their performance over using only EHG parameters (i.e., RF_F1_1_ vs. RF_F1_3_ and RF_F1_2_ vs. RF_F1_4_) without statistically significant differences between them (except in specificity RF__F1_2_ vs. RF__F1_4_). On the other hand, the use of the 10th–90th percentiles vs. 50th percentile of the EHG parameters as inputs (i.e., RF_F1_1_ vs. RF_F1_2_ and RF_F1_3_ vs. RF_F1_4_) reduced F1-score and sensitivity, with no statistically significant differences. There were only statistically significant differences in terms of specificity between the RF__F1_4_ and the rest with a mean specificity value of 93.75 ± 5.97%, whereas the mean specificity values for the other RF classifiers range from 97.78 ± 4.2% to 98.75 ± 2.48%. RF metrics for the validation dataset showed high mean values for specificity, but modest sensitivity (from 73.83 ± 12.08% to 81.83 ± 12.9%). The mean values of the RFs metrics for the test group are shown in Figure 2. As occurs in the validation group, the highest F1-score for the test belongs to RF_F1_2_, but dropping its values to 80.35 ± 6.78%. The RF classifiers stand out especially for their high specificity metrics (over 90% for all test datasets), but with low sensitivities, ranging from 65.78 ± 11.61% to 74 ± 10.41%, this latter for RF_F1_2_. The high variability of the RF classifier metrics is also noticeable in the test group (Table 3), especially for sensitivity, with coefficients of variation between 14.1% and 17.6%, which is a major drawback in predicting preterm labor.

### 3.2. Extreme Learning Machine (ELM)

**Figure 3:**
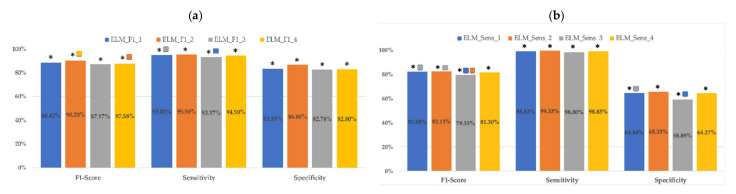
Mean values of different ELM classifier metrics for validation datasets in the 30 data partitions (**a**) optimizing F1-score (**b**) optimizing sensitivity. For each optimization criteria and metric, the significant differences (*p* < 0.05) for each input dataset are marked with 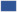 10th–90th percentiles of EHG parameters + obstetric input data; 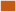 50th percentile of EHG + obstetric input data; 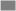 10th–90th percentiles of EHG parameters; 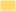 50th percentile of EHG parameters. Significant differences between the two optimization criteria for the same input data set are marked with *.

Figure 3 shows a bar plot of the metrics of the ELM classifiers for each set of input parameters in the validation dataset when optimizing with F1-score and sensitivity. First of all, it should be noted that optimizing ELM classifiers with the F1-score or sensitivity criteria resulted in statistically significant differences (ELM_F1_X_ vs. ELM_SEN_X_) in all their metrics for the same input dataset. ELM_F1_ outperforms ELM_SEN_ classifiers in F1-score and specificity metrics, but gives lower values for sensitivity. This latter increase in ELM_SEN_ sensitivity metrics (about 3.5%) was at the cost of a notable reduction in specificity (about 20%). For instance, comparing ELM_Sen_2_ and ELM_F1_2_, an improvement of around 4% (95.5 + 4.61% vs. 99.33 + 1.73) in sensitivity led to a reduction of more than 20% in specificity (86.8 + 7.14% vs. 65.33 + 10.78%) and to a statistically significant reduction in the F1-score from 90.2 + 4.43% to 82.11 + 4.5%. It should also be noted that, unlike what happened with the RF classifiers, the ELM_F1_ classifiers presented high sensitivity values, with mean values between 93.17 ± 5% and 95.5 ± 4.61% for the validation dataset. This performance is of special importance when developing imminent labor predictive systems in women with TPL and for preterm labor prediction in general

Analyzing the effect of the input features in the classifier performance, regardless of the optimization criteria, the highest F1-score was reached by the classifiers that used the 50th percentile of EHG parameters and obstetric data, ELM_F1_2_ (90.2 ± 4.43%) and ELM_SEN_2_ (82.11 ± 4.5%). ELM_F1_2_ and ELM_SEN_2_ also reported the highest sensitivities (95.5 ± 4.61% and 99.33 ±1.73%) and specificities (86.8 ± 7.14% and 65.33 ± 10.78%) for each optimization criteria. For the same optimization criteria, these metrics did not present statistically significant differences with those of classifiers using the 10th–90th percentile of EHG parameters and obstetric data as inputs (ELM_X_1_ vs. ELM_X_2_). Using only EHG parameters as data inputs slightly worsens ELM classifier metrics compared to the combined use of EHG and obstetric data (ELM_X_1_ vs. ELM_X_3_ and ELM_X__ vs. ELM_X_4_).

On the other hand, the performances of the ELM classifiers for the test datasets are consistent with those obtained in the validation dataset, although all the metrics are reduced (see Table 4). The highest F1-score, sensitivity and specificity were reached by ELM_F1_2_ with corresponding 82.14 ± 5.88%, 89.89 ± 7.14% and 76.4 ± 8.12% values. Similarly, when optimizing by sensitivity criteria, the highest F1-score, sensitivity and specificity values for the test dataset are for ELM_SEN_2_, but dropping its F1-score to 75.42 ± 3.96%, mainly due to the low specificity of 52.25 ± 9.58%, regardless of the high sensitivity of 96.00 ± 5.13%. Indeed, the results of the ELM classifiers for test datasets reveal that specificity has the greatest variability, especially when the sensitivity optimization criterion is applied, reaching 19.5% (ELM_SEN_4_) in this case.

### 3.3. K-Nearest Neighbors (KNN)

**Figure 4:**
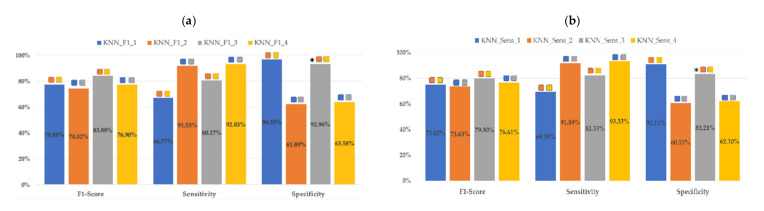
Mean values of different KNN classifier metrics for validation datasets in the 30 data partitions: (**a**) optimizing F1-score (**b**) optimizing sensitivity. For each optimization criteria and metric, the significant differences (*p* < 0.05) for each input dataset are marked with 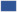 10th–90th percentiles of EHG parameters + obstetric input data; 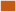 50th percentile of EHG + obstetric input data; 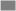 10th–90th percentiles of EHG parameters; 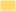 50th percentile of EHG parameters. Significant differences between the two optimization criteria for the same input dataset are marked with *.

As can be seen in Figure 4, KNN classifier metrics do not present statistically significant differences according to the optimization criteria, except in the case of the specificity between KNN_F1_3_ and KNN_SENS_3_. The highest F1 score in the validation dataset for each optimization criteria is for KNN_F1_3_ (83.88 ± 10.31%) and KNN_SENS_3_ (79.9 ± 9.72%) with the 10th–90th percentiles of EHG parameters as inputs.

As regards the influence of the classifier input dataset on their performance, KNN_F1_3_ did not present statistically significant differences in any of its metrics with respect to KNN_F1_1_. The same occurred with KNN_F1_2_ vs. KNN_F1_4_. That is, having added obstetric inputs did not improve KNN classifier metrics. However, using the 50th or 10th–90th percentiles of EHG gave significant differences in all KNN metrics for the validation dataset. For instance, KNN_F1_1_ showed statistically higher specificity than KNN_F1_2_ (96.45 ± 3.99% vs. 61.89 ± 12.02%) and lower sensitivity (66.77 ± 13.85% vs. 91.53 ± 7.92%). This also occurred in KNN_F1_3_ and KNN_F1_4_. It should be noted that the highest mean sensitivity was reached by KNN_F1_4_ (92.83 ± 6.11%) without significant differences with KNN_F1_2_ (91.53 ± 7.92%) but at the cost of a considerable reduction in specificity, with corresponding values of 63.58 ± 10.57% and 61.89 ± 12.02%. As previously mentioned, the same behavior was observed in KNN_Sens_ classifiers: the use of 10th–90th or 50th percentiles of EHG parameters modified KNN_SENS_ performance with significant differences in all metrics (see Figure 4). Sensitivity is greater when using the 50th percentile and F1-score, and specificity when using the 10th–90th percentiles. The inclusion of obstetric inputs did not improve KNN_SENS’_ metrics either.

Mean values for the KNN metrics for test dataset are summarized in Table 5 and are consistent with those from the validation dataset: in this case, the optimization criteria caused noticeable differences in classifier metric values for the same data input, but with similar tendencies. For F1-score optimization, the highest F1-score corresponded to KNN_F1_3_ (84.67 ± 8.46%) and was similar to that of KNN_F1_1_ (84.18 ± 9.47%), associated with high specificity metrics (93.42 ± 6.34% and 92.7 ± 8.81%) and moderate sensitivities (79.33 ± 13.23% and 80.56 ± 12.57%). For sensitivity optimization, the highest F1-score corresponded to KNN_SENS_1_ (79.8 ± 8.29%), similar to that of KNN_SENS_3_ (78.63 ± 8.6%) and was lower than that of F1-score optimization criteria. In this case, for the test group the greatest variability in F1-score and sensitivity are associated with classifiers with input parameters that use the P10–90 percentiles of the EHG parameters, while in the case of specificity it is for those that use the 50th percentile as inputs. Indeed, for the test dataset, the use of the 50th percentile of EHG parameters improved sensitivity for the test dataset while dramatically reducing specificity when using sensitivity optimization criteria.

### 3.4. Comparison of Classifiers

**Figure 5:**
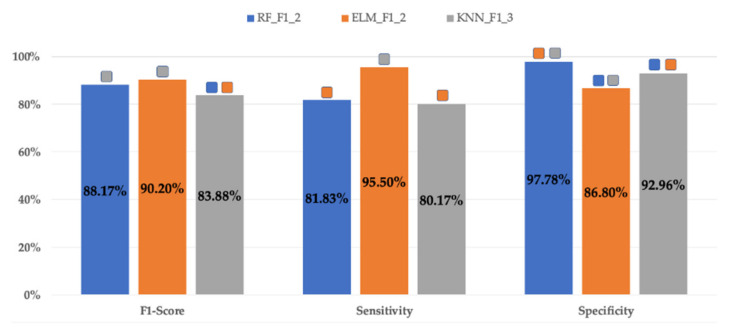
Mean values of different classifier metrics for validation datasets in the 30 data partitions obtained for the best RF, ELM and KNN classifiers. Significant differences (*p* < 0.05) of the classifiers and metrics with the others are marked with 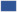 RF_F1_2_; 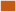 ELM_F1_2_; 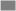 KNN_F1_2_.

The metrics for RF, ELM and KNN classifiers with the best performance (best F1-score in validation dataset) are shown in Figure 5. All of them corresponded to F1-score optimization criteria. ELM_F1_2_ achieved the highest F1-score (90.2 ± 4.43%) with statistically significant differences with KNN_F1_3_ (83.88 ± 10.31%) but not with RF_F1_2_ (88.17 ± 8.34%). RF_F1_2_ and ELM_F1_2_ presented statistically significant differences in terms of sensitivity and specificity, the sensitivity being highest for ELM_F1_2_ (95.5 ± 4.61% vs. 81.83 ± 12.9%) and the specificity for RF_F1_2_ (97.78 ± 4.2% vs. 86.8 ± 7.14%). Apart from having shown the lowest F1-score, KNN_F1_3_ showed the lowest sensitivity (80.17 ± 15.17%) and statistically lower specificity (92.96 ± 5.86%) than RF_F1_2_.

**Figure 6:**
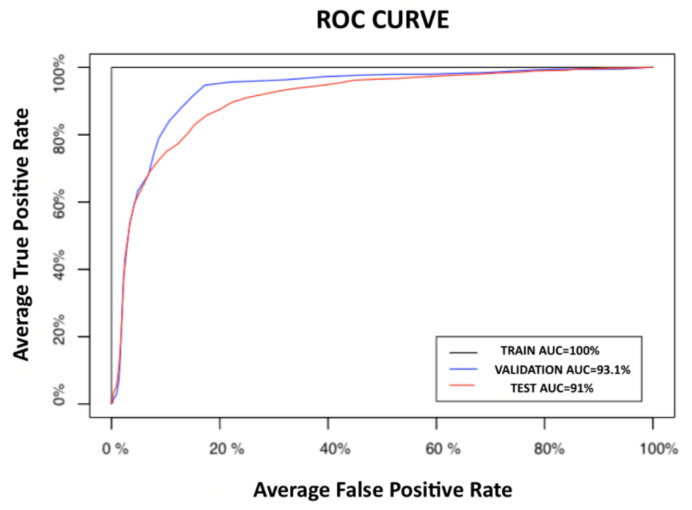
Average receiver operating curves (ROCs) for training, validation and test datasets for the ELM_F1_2_.

Bearing in mind that in the clinical scenario for the application of these classifiers, the prediction of preterm delivery, a false positive diagnosis is preferable to stopping treating a false negative (premature that has been identified as a false threat), the classifier with the best performance was the ELM_F1_2_, that is the one that makes a combined use of obstetric and the 50th percentile of EHG parameters. Figure 6 shows the average ROC curves for the ELM_F1_2_ classifier, with an AUC of 93.1% for the validation dataset and 91.0% for the test dataset.

## 4. Discussion

Although several studies deal with the use of EHG for preterm labor prediction in women recorded during regular checkups in a drug-free physiological state [21,22,31,32,34,37,52], the literature is scarce on preterm labor predictive systems in women with TPL under the effect of tocolytic drugs [20], even though tocolytic drugs are usually clinically administered at the first signs of TPL. These drugs were found to modify the EHG characteristics and these changes are dependent on the phase of the drug administration in which the recordings were made [25,26]. Despite this, the usefulness of EHG for the prediction of imminent delivery in women with TPL under tocolytic treatment has already been checked in a previous study using ANN [27]. In the present work we aimed to overcome some limitations of that study, such as the low learning speed associated with ANN backpropagation, by using computationally efficient algorithms such as RF, ELM and KNN. We selected a random forest algorithm, which uses an ensemble of decision tree classifiers, because it is easy to implement and there are reports that it provides a better performance than other classification algorithms, such as ANN [53]. The ELM, a feedforward neural network with a single-hidden layer, accelerates the running speed of the identification model. ELM has been shown to be more stable than ANN, with lower variance of its metrics, and is more suitable for real-time applications in situations that require rapid reactions [54]. ELM has been used in obstetrics to identify labor and nonlabor contractions from EHG recordings [28]. Finally, KNN, a nonparametric and therefore low complex algorithm previously used in EHG classification [55,56], was also assessed in the present work. We studied how the optimization criteria used for the classifiers affected their performance, which has not been clearly indicated in most published studies, regardless of the classification algorithm employed [24,29,31]. We proposed two optimization criteria: F1-score and sensitivity. It is noteworthy that when using sensitivity as optimization criterion the optimal hyperparameters were considered those that provided the best average F1-score in the validation dataset so as to reach a trade-off between sensitivity and specificity. Otherwise, the option is to consider that all women with TPL will deliver prematurely and will therefore require tocolytic drugs and lung maturation corticosteroids, which is a widespread clinical practice nowadays.

Analyzing the influence of the two optimization criteria proposed—F1-score and sensitivity—RF resulted in a unique optimal RF structure, which did not occur for ELM and KNN classifiers, which could have been caused by the nonlinear hyperparameters involved as activation functions and kernels in ELM and KNN classifiers. For ELM, classifiers designed to optimize sensitivity achieved a slight improvement in their sensitivity metrics compared to optimizing the F1-score, at the cost of a decreasing specificity and F1-score. Indeed, in the case of ELM, for both optimization criteria (F1-score and sensitivity) the sensitivity metrics always exceed those of specificity, with values over 90% in validation and 86% in test. This behavior, not observed in KNN, is especially appropriate in the design of imminent labor predictive systems in women with TPL. On the other hand, the KNN metrics did not show statistically significant differences between both optimization criteria for the same input dataset.

The highest F1-score value in validation dataset were obtained for RF_F1_2_ (88.17 ± 8.34%) and ELM_F1_2_ (90.2 ± 4.43%), both with the 50th percentile of EHG and obstetric data inputs. They also showed the highest sensitivity (81.83 ± 12.9% and 95.5 ± 4.61%). The good performance of RF metrics agrees with previous studies. Idowu et al. analyzed the performance of several machine learning algorithms for preterm labor detection using the TPEHG database from Physionet and found that random forest performed the best, with a specificity of 86%, sensitivity of 97%, and AUROC of 94% compared with penalized logistic regression and a rule-based classifier [57]. Ren et al. compared the performance of several classifiers based on EHG signals from the TPEHG Physionet database (routine checkups) to differentiate term and preterm deliveries. They used empirical mode decomposition to obtain Intrinsic Mode Functions and then entropy values, and found that RF and AdaBoost outperformed support vector machine, multilayer perceptron, Bayesian network, and simple logistic regression [31].

We consider that ELM_F1_2_ provides a better performance than RF_F1_2_ due to its higher sensitivity, which is decisive in this application, as previously mentioned. In this regard, Chen and Hao developed an ELM classifier based on EHG to differentiate labor and nonlabor contractions, manually segmented from the PhysioNet Icelandic 16-electrode Electrohysterogram Database, also reporting high sensitivity metrics [28]. Chen et al. assessed the performance of stacked sparse autoencoder (SSAE), SVM and ELM to identify labor contractions using the Icelandic Database [30], obtaining a slightly better performance for SSAE but without carrying out statistical tests. ELM_F1_2_ metrics are slightly higher than those previously obtained using the same EHG recording database with an ANN classifier, for both validation and test groups (F1-score of 84.3 ± 5.0%, sensitivity of 86.5 ± 7.4% and specificity of 81.5 ± 7.3 for the validation dataset with ANN and F1-score of 80.3 ± 5.5%, sensitivity of 81.6 ± 9.4% and specificity of 78.8 ± 5.8% for the test dataset) [27]. Indeed, AUC values for ELM_F1_2_ in validation and test were over 93%, similar to those reported in the literature for preterm labor predictive systems based on EHG recordings during regular checkups [12,21,22,31,37,55,58], and slightly higher than those achieved in imminent labor prediction in women with TPL using ANN (AUC validation 91.8 ± 3.2%, AUC test 87.1 ± 4.3%) [27]. This could be due to the fact that in order to avoid overfitting, the two optimization criteria were applied to the validation dataset, whereas in our previous work the square root of the training and validation F1-score was optimized [27].

The F1-score and sensitivity metrics of the KNN classifiers underperformed RF and ELM in the validation dataset. These results are consistent with those obtained by Fergus et al. when using different classifiers to distinguish between preterm and term birth with the TPEHG database, without a test group but with cross validation [55]. KNN provided worse results than decision trees and polynomial classifiers. Indeed KNN is greatly reliant on the input features’ dimensionality and the training dataset [40,43], resulting in lower values for its metrics [59,60]. However, in the present study KNN classifiers showed a high generalization capability with very similar metrics between validation and test.

With reference to the discriminatory capacity of the classifiers depending on the four different input data sets, the 50th or 10th–90th percentiles of EHG parameters with or without obstetric parameters, different outcomes were observed. In general, the RF classifier metrics were little influenced by the input dataset, although the use of obstetric parameters seems to slightly improve their specificity. This is in agreement with previous studies: Obstetric parameters such as cervical length or fetal fibronectin show high negative predictive but low positive predictive capabilities [8,61]. ELM algorithms also presented very similar metrics for the four sets of proposed input parameters when using the same optimization criterion. In fact, when only EHG characteristics were used, there were no differences in any of the ELM classifier metrics and adding obstetric data improved specificity. On the other hand, the KNN classifier metrics were highly dependent on the input dataset. The best F1-score in the validation dataset for KNN was obtained for KNN_F1_3_ and KNN_Sens_3_ (83.88 ± 10.31% and 79.9 ± 9.72%, respectively) for both optimization criteria, with the 10th–90th percentiles of EHG and obstetric data inputs. These results agree with Mas-Cabo et al., who obtained a higher discrimination capability between term and preterm births in the 10th and 90th percentiles of EHG parameters in women recorded during routine checkups [20].

Despite the good results obtained, the present study still has certain shortcomings. Firstly, a larger database would be more representative of the target population and would further corroborate the performance of this imminent labor prediction system. Increasing the database would also allow contextualization of the EHG records, allowing the phase of tocolytic treatment in which they were obtained to be considered, since previous works revealed a significant effect of this drug on the EHG parameters, especially on spectral and nonlinear ones [25]. Secondly, even with a larger database we would have to deal with inter-class data imbalance. In the present work there were about 25% fewer women delivering ≤7 days than >7 days, which is in agreement with preterm prevalence in women with TPL [62]. The SMOTE oversampling technique was used here to tackle this problem. Weighted classifiers or boosting ensemble learning could bring about more reliable prediction systems. Thirdly, the use of PCA to reduce the input parameters’ dimensionality makes it difficult to discern which of them are the best to discriminate imminent labor in women with TPL without considering nonlinear relationships, which are often present in biological systems [63]. In future work we aim to use other feature selection techniques that will allow us to determine an optimized feature subset, such as random forest or particle swarm optimization, among others [64,65], to develop low complexity classifiers that are easy to understand with improved metrics. Finally, a robust algorithm to automatically remove artefacted EHG signals or identify EHG-Bursts before feature extraction would help the development of imminent labor prediction systems for clinical practice. Even though some studies have already been carried out on this [66,67,68,69], it is still one of the main obstacles that prevents the clinical use of EHG

## 5. Conclusions

The present work confirms that it is possible to predict imminent labor in women with TPL undertaking tocolytic treatment by computationally efficient algorithms based on EHG and obstetric parameters. RF and ELM with the 50th percentile of EHG and obstetric input parameters provided the highest F1-score values for the validation dataset, but ELM outperformed RF sensitivity metrics. The use of the 10th–90th percentiles did not result in a significant improvement of these classifier metrics over the 50th percentile. As for the two optimization criteria analyzed for classifiers’ design (F1-score and sensitivity), RFs and KNN were barely affected, but for ELM optimizing sensitivity slightly increases sensitivity compared to optimizing F1-score, but seriously reduces specificity and therefore F1-score. KNN classifier performance was highly sensitive to the input dataset and the test metrics revealed a high generalization capability.

## Figures and Tables

**Table 1:** Summary of electrohysterographic recordings (EHG) features and obstetric data inputs.

EHG Temporal Parameters	EHG Spectral Parameters	EHG Nonlinear Parameters	Obstetric Data
Peak-to-peak amplitude	DF1 DF2 H/L ratio Deciles [D1–D9] SMR	Binary Lempel-Ziv Multistate Lempel-Ziv (n = 6) Sample entropy Spectral entropy Fuzzy entropy Time reversibility SD1 SD2 SD1/SD2	Cervical length Gestational age at moment of recording Maternal age Gestations Parity Abortions

**Table 2:** Summary of the classifiers developed, their input features and optimization criterion (F1-score or sensitivity). RF: random forest, ELM: extreme learning machine, K-nearest neighbors (KNN).

		RF		ELM		KNN	
	Criterion	F1-Score	Sensitivity	F1-Score	Sensitivity	F1-Score	Sensitivity
Input Features							
EHG 10th–90th percentiles + Obstetric data		RF_F1_1_	RF_SEN_1_	ELM_F1_1_	ELM_SEN_1_	KNN_F1_1_	KNN_SEN_1_
EHG 50th + Obstetric data		RF_F1_2_	RF_SEN_2_	ELM_F1_2_	ELM_SEN_2_	KNN_F1_2_	KNN_SEN_2_
EHG 10th–90th percentiles		RF_F1_3_	RF_SEN_3_	ELM_F1_3_	ELM_SEN_3_	KNN_F1_3_	KNN_SEN_3_
EHG 50th percentile		RF_F1_4_	RF_SEN_4_	ELM_F1_4_	ELM_SEN_4_	KNN_F1_4_	KNN_SEN_4_

**Table 3:** Mean ± standard deviation and coefficient of variation (in brackets) of RF classifiers performance metrics in test dataset for predicting imminent birth (TTD ≤ 7days) in women with threatened preterm labor (TPL) using EHG data or a combination of EHG and obstetric data. The maximum value for each metric is shown in bold. F1: F1-score, Sens: Sensitivity, Spec: Specificity.

Opt. Criterion	Inputs	Classifier	Test_F1	Test_Sens	Test_Spec
F1-Score Sensitivity	EHG_P10–P90_ + Obs	RF_F1_1_	77.51 ± 7.58% (9.8%)	66.22 ± 11.70% (17.7%)	97.12 ± 4.13% (4.3%)
	EHG_P50_ + Obs	RF_F1_2_	**80.35 ± 6.78% (8.4%)**	**74.00 ± 10.41% (14.1%)**	92.25 ± 5.35% (5.8%)
	EHG_P10–P90_	RF_F1_3_	77.81 ± 8.71% (11.2%)	65.78 ± 11.61% (17.6%)	**98.29 ± 2.51% (2.6%)**
	EHG_P50_	RF_F1_4_	77.7 ± 6.6% (8.5%)	71.44 ± 10.99% (15.4%)	90.72 ± 4.58% (5.0%)

**Table 4:** Mean ± standard deviation and coefficient of variation (in brackets) of ELM classifiers’ performance metrics in test dataset for predicting imminent birth (TTD ≤ 7 days) in women with TPL using EHG data or a combination of EHG and obstetric data. The maximum value for each metric and optimization criterion is shown in bold. F1: F1-score, Sens: sensitivity, Spec: specificity.

Opt. Criterion	Inputs	Classifier	Test_F1	Test_Sens	Test_Spec
F1-score	EHG_P10–P90_ + Obs	ELM_F1_1_	80.00 ± 4.98% (6.0%)	87.56 ± 8.53% (9.7%)	74.77 ± 7.32% (9.8%)
	EHG_P50_ + Obs	ELM_F1_2_	**82.14 ± 5.88% (7.2%)**	**89.89 ± 7.14% (7.9%)**	**76.40 ± 8.12% (10.6%)**
	EHG_P10–P90_	ELM_F1_3_	78.41 ± 4.55% (5.8%)	85.89 ± 7.91% (9.2%)	73.24 ± 6.93% (9.5%)
	EHG_P50_	ELM_F1_4_	79.00 ± 5.06% (6.4%)	86.22 ± 6.65% (7.7%)	73.87 ± 8.64% (11.7%)
Sensitivity	EHG_P10–P90_ + Obs	ELM_SEN_1_	74.83 ± 3.88% (5.2%)	95.44 ± 4.59% (4.8%)	51.35 ± 9.28% (18.1%)
	EHG_P50_ + Obs	ELM_SEN_2_	**75.42 ± 3.96% (5.3%)**	**96.00 ± 5.13% (5.3%)**	**52.25 ± 9.58% (18.3%)**
	EHG_P10–P90_	ELM_SEN_3_	73.13 ± 3.10% (4.2%)	94.78 ± 4.61% (4.9%)	47.57 ± 8.83% (18.6%)
	EHG_P50_	ELM_SEN_4_	73.83 ± 3.24% (4.4%)	94.89 ± 5.01% (5.3%)	49.37 ± 9.63% (19.5%)

**Table 5:** Mean ± standard deviation and coefficient of variation (in brackets) of KNN classifier performance metrics in test dataset for predicting imminent birth (TTD ≤ 7 days) in women with TPL using EHG characteristics or a combination of EHG and obstetric data. The maximum value for each metric and optimization criterion is in bold. F1: F1-score, Sens: Sensitivity, Spec: Specificity.

Opt. Criterion	Inputs	Classifier	Test_F1	Test_Sens	Test_Spec
F1-score	EHG_P10–P90_ + Obs	KNN_F1_1_	84.18 ± 9.47% (11.2%)	79.33 ± 13.23% (16.7%)	**93.42 ± 6.34% (6.8%)**
	EHG_P50_ + Obs	KNN_F1_2_	74.16 ± 5.07% (6.8%)	**93.33 ± 6.37% (6.8%)**	52.43 ± 9.59% (18.3%)
	EHG_P10–P90_	KNN_F1_3_	**84.67 ± 8.46% (10.0%)**	80.56 ± 12.57% (15.6%)	92.70± 8.81% (9.5%)
	EHG_P50_	KNN_F1_4_	74.13 ± 4.57% (6.2%)	90.89 ± 6.55% (7.2%)	55.77 ± 9.67% (17.3%)
Sensitivity	EHG_P10–P90_ + Obs	KNN_SEN_1_	**79.8 ± 8.29% (10.4%)**	82.78 ± 12.13% (14.7%)	**80.36 ± 9.76% (12.1%)**
	EHG_P50_ + Obs	KNN_SEN_2_	72.98 ± 4.00% (5.5%)	**94.22 ± 5.67% (6.0%)**	47.93 ± 8.98% (18.7%)
	EHG_P10–P90_	KNN_SEN_3_	78.63 ± 8.60% (10.9%)	83.56 ± 12.47% (14.9%)	76.58 ± 14.2% (18.5%)
	EHG_P50_	KNN_SEN_4_	73.19 ± 4.31% (5.9%)	91.78 ± 7.15% (7.8%)	52.07 ± 9.39% (18.0%)

## Data Availability

The data that support the findings of this study are available on request from the corresponding author. The data are not publicly available due to contain information that could compromise the privacy of participants.

## Appendix A

**Table A1:** Hyperparameters optimized for each classifier and gridsearch carried out (in brackets).

RF Hyperparameters	ELM Hyperparameters	KNN Hyperparameters
Number of trees (100, 200, 500, and 750)	Number of neurons in the hidden layer (100, 500, 750, 1000, 2000, and 30,000);	Number of neighbors (1, 3, 5, and 7)
Maximum depth of these trees (6, 10, and unlimited)	Activation function (hyperbolic tangent and sigmoid).	Kernel used for weighting the distances (triangular, Biweight and Epanechnikov).
Cost of division based on the criterion of gain of information were optimized (0.001, 0.2, and 0.5)		

**Table A2:** Hyperparameters’ combination for the optimal RF classifiers in validation.

Opt. Criterion	Inputs	Classifier	Number of Neurons	Activation Function
F1-score	EHG_P10–P90_ + Obs	ELM_F1_1_	500	Sigmoid
	EHG_P50_ + Obs	ELM_F1_2_	500	Sigmoid
	EHG_P10–P90_	ELM_F1_3_	500	Sigmoid
	EHG_P50_	ELM_F1_4_	500	Sigmoid
Sensitivity	EHG_P10–P90_ + Obs	ELM_SEN_1_	750	Sigmoid
	EHG_P50_ + Obs	ELM_SEN_2_	1000	Sigmoid
	EHG_P10–P90_	ELM_SEN_3_	750	Sigmoid
	EHG_P50_	ELM_SEN_4_	500	Sigmoid

**Table A3:** Hyperparameters’ combination for the optimal ELM classifiers in validation.

Opt. Criterion	Inputs	Classifier	Number of Neurons	Activation Function
F1-score	EHG_P10–P90_ + Obs	ELM_F1_1_	500	Sigmoid
	EHG_P50_ + Obs	ELM_F1_2_	500	Sigmoid
	EHG_P10–P90_	ELM_F1_3_	500	Sigmoid
	EHG_P50_	ELM_F1_4_	500	Sigmoid
Sensitivity	EHG_P10–P90_ + Obs	ELM_SEN_1_	750	Sigmoid
	EHG_P50_ + Obs	ELM_SEN_2_	1000	Sigmoid
	EHG_P10–P90_	ELM_SEN_3_	750	Sigmoid
	EHG_P50_	ELM_SEN_4_	500	Sigmoid

**Table A4:** Hyperparameters’ combination for the optimal KNN classifiers in validation.

Opt. Criterion	Inputs	Classifier	Number of Neighbors	Kernel
F1-score	EHG_P10–P90_ + Obs	KNN_F1_1_	2	Triangular
	EHG_P50_ + Obs	KNN_F1_2_	7	Biweight
	EHG_P10–P90_	KNN_F1_3_	2	Triangular
	EHG_P50_	KNN_F1_4_	7	Biweight
Sensitivity	EHG_P10–P90_ + Obs	KNN_SEN_1_	7	Triangular
	EHG_P50_ + Obs	KNN_SEN_2_	7	Epanechnikov
	EHG_P10–P90_	KNN_SEN_3_	5	Triangular
	EHG_P50_	KNN_SEN_4_	7	Triangular
